# *CRISPLD2* Gene Polymorphisms with Nonsyndromic Cleft Lip Palate in Indian Population

**DOI:** 10.1055/s-0040-1713166

**Published:** 2020-07-08

**Authors:** Praveen Kumar Neela, Srinivas Reddy Gosla, Akhter Husain, Vasavi Mohan

**Affiliations:** 1 Department of Orthodontics, Yenepoya University, Mangaluru, Karnataka, India; 2 Department of Craniofacial Surgery, GSR Institute of Craniofacial Surgery, Hyderabad, Telangana, India; 3 Department of Genetics, Vasavi Medical Research Centre, Hyderabad, Telangana, India

**Keywords:** cleft lip palate, gene, polymorphism, *CRISPLD2*, MassARRAY

## Abstract

Cleft lip palate (CLP) is one of the common congenital anomalies with multifactorial etiology. Many genes are associated with its etiology. In one of the studies *CRISPLD2* gene polymorphisms rs1546124, rs4783099, and rs16974880 were reported in the Chinese population. However, its role in the Indian population is not yet studied. Hence, this research was conducted on the Indian population to know the role of these high-risk polymorphisms in patients with nonsyndromic CLP. Following an inclusion and exclusion criteria, 20 multiplex CLP families were selected from a high volume cleft center in India. Genomic DNA was isolated from these families. Single nucleotide polymorphism (SNP) rs1546124, rs4783099, and rs16974880 were analyzed for their association using MassARRAY method. A whole-genome association analysis toolset, PLINK was used for statistical analysis. The polymorphisms followed Hardy–Weinberg equilibrium. None of the polymorphisms showed any significance. Hence the high-risk polymorphisms rs1546124, rs4783099, and rs16974880 are not associated with nonsyndromic CLP in Indian population.

## Introduction

Cleft lip palate (CLP) is an important congenital disability affecting humans. An infant is born with a cleft lip and/or palate somewhere on the planet every 2 minutes according to a World Health Organization (WHO) study. 1 Prevalence of cleft lip and palate varies significantly from one country to another. It is highest in North American Indians and East Asians (1:2,500) and lowest in Africans (1:500). The cleft incidence in India is around 1:800 to 1:1,000, and three infants are born with some type of cleft every hour. 2 Cleft lip palate can be syndromic or nonsyndromic. A total of 70% of the cleft lip and palate cases are nonsyndromic, whereas 30% are syndromic which are associated with some other anomalies. 3 Our understanding of the etiology and pathogenesis of nonsyndromic variants yet remains relatively poor. The etiology is multifactorial, ranging from genetic causes, malnutrition, endocrine disorders, infection, trauma, consanguinity, etc. Roughly 20% of the CLP showed consanguinity of their parents while the percentage of familial cases is 3.5% of all the cleft cases. 4 Some form of cleft phenotype characterizes approximately 600 syndromes. 5

### Genetic Causes

Genetic research of clefts uses both association analysis and link analysis to determine the genetic determinants. The results of candidate gene-based association studies, performed on various ethnicities, populations have been mostly inconclusive or conflicting, with many candidate loci implicated in cleft phenotypes. Inconsistency is mostly due to genetic heterogeneity. Various researchers discovered multiple candidate genes linked to nonsyndromic CLP (NSCLP) such as IRF6, MSX1, ABC4, RARA, TGFα, TGFβ, p63, MYH9, BCL3, MTHFR, TGFB2, SATB2, P63, MSX2, FOXE1, BMP4, PAX7, PVRL1, TGFB3, RARA, RUNX2, BCL3, TGFB1, TBX1, and BCL3. 6 7 8 9 10 11 12 13 14 Genetic variation in cysteine-rich secretory protein **L** imulus clotting factor **C** , **C** ochlin (Coch-5b2) and **L** gl1 (LCCL) domain containing 2 ( *CRISPLD2* ) gene reported as an etiological factor in CLP. 15 Three SNPs identified in the study analyzed for its association in Northern Chinese population revealed significant association. 16 Studies conducted later also reported the involvement of this gene. 17 18 Even studies on animal models also showed that it is an important gene associated with NSCLP. 19

However, there is no reported data on the association of these SNPs in the Indian population. Therefore, the present study was aimed to evaluate the role of these high-risk SNPs in the etiology of CLP in Indian multiplex families of NSCLP.

## Materials and Methods

### Ethical Approval of Research

The Institutional Review Board of GSR Institute of Craniofacial Surgery, Hyderabad, India, approved this study. It is a high-volume cleft center in India. Multiplex families of Indian origin with NSCLP were selected. Patients with a monogenic syndrome or chromosomal aberrations, associated malformations, and mental retardation were excluded from the study. The control sample comprised of unaffected related individuals from these multiplex families. Based on the power calculation for family-based association studies, 20 multiplex families were selected. These include 1 family with five affected, 2 families with four affected, 5 families with three affected, and 12 families with two affected cleft patients. Four multigenerational families reported consanguinity. Informed consent obtained from all the participants. Parents' consent was taken when the affected participants were minors.

### DNA Isolation

Venous blood of 4 to 5 mL was taken in the Ethylene Diamine Tetra Acetic acid (EDTA) tubes. Genomic DNA was extracted from the blood lymphocytes using the salting-out method. 20 An ultraviolet spectrometer was used to calculate the average 260/280 nm ratio to assess the purity and concentration of DNA. The ratio of absorbance readings at the two wavelengths should be between 1.8 and 2.0 (i.e., A260/A280 = 1.7–2.0). Later, the DNA sent for SNP genotyping of the polymorphisms.

### Characteristics of the Single Nucleotide Polymorphism

The characteristics of the selected polymorphisms rs1546124, rs4783099, and rs16974880 ( Table 1 ) are taken from web sites ( www.ncbi.nlh.nih.gov/snp/ and http://asia.ensembl.org/Homo_sapiens/Info/Index ).

**Table 1:** Characteristics of nucleotide variants selected for the study

Gene	Polymorphism	Type of alteration	Alleles	Ancestral allele	Global MAF
*CRISPLD2*	rs1546124	5 Prime UTR variant	G/A/C	C	0.34
*CRISPLD2*	rs4783099	3 Prime UTR variant	C/T	C	0.39
*CRISPLD2*	rs16974880	3 Prime UTR variant	T/A/G	T	0.24

Abbreviations: A, adenine; C, cytosine; G, guanine; MAF, minor allele frequency; T, thymine; UTR, untranslated region.

### Single Nucleotide Polymorphism Genotyping

The SNP genotyping was done utilizing the Agena Bio MassARRAY (Agena Bioscience, Inc.; San Diego, California, United States) platform using iPLEX Gold technology. This platform is a nonfluorescent, highly accurate detection platform utilizing Matrix-Assisted Laser Desorption/Ionization—Time of Flight (MALDI-TOF) mass spectrometry. The assay was designed using proprietary Agena software (Assay Design Suite 2.0). The assay design was used to design primers. Follow the correct workflow according to the MassARRAY protocol, and finally run the sample through the analyzer. Agena's Spectro Typer 4.0 software (San Diego, California, United States) was used, which automatically generates reports that identify the SNP alleles (homozygous or heterozygous). The data obtained from the analyzer software is sent for statistical analysis.

### Data Analyses

The SNP allele data of the affected and unaffected obtained from the MassARRAY system was subjected to statistical analysis. PLINK software (version 1.09) was used for this study. 21 It is an open-source whole-genome association toolset, which performs a varied range of analyses from basic-to-large scale. Genotype distribution was used to calculate the Hardy–Weinberg equilibrium (HWE). Comparisons between the affected and unaffected were performed using this PLINK software. Odds ratio (OR) and 95% confidence intervals (CIs) were provided. Allelic association was analyzed using the Chi-square test. For nominal association, the statistical significance level is set to *α* = 0.05

## Results

All the three SNPs present on *CRISPLD2* were genotyped in 20 multiplex families. All the polymorphisms follow Hardy–Weinberg equilibrium. In the allele association analysis ( Table 2 ), we observed that none of the variants showed any association with NSCLP. None of them showed a *p* -value of < 0.05. The OR also was less than 1.3.

**Table 2:** Association between *CRISPLD2* polymorphisms and NSCLP

SNP	BP	A1	F_A	F_U	A2	CHISQ	*p* -Value	OR (95% CI)
rs1546124	16	G	0.17	0.1974	C	0.2174	0.641	0.84
rs4783099	17	T	0.27	0.2237	C	0.494	0.4821	1.29
rs16974880	24	A	0.12	0.1184	T	0.001024	0.9745	1.01

Abbreviations: A, adenine; A1, major allele (wild allele); A2, minor allele (mutant); BP, base pairs; CHISQ, Chi-square; CHR, chromosome number; CI, confidence interval; F_U, minor allele frequency unaffected; F_A, minor allele frequency affected; G, guanine; NSCLP, nonsyndromic cleft lip plate; OR, odds ratio; SNP, single nucleotide polymorphism; T, thymine.

Note: the *p* -value < 0.05 is significant.

## Discussion

Development of craniofacial complex is one of the complex events during early phases of embryonic development, coordinated by a network of various transcription factors, and signaling molecules along with proteins conferring cell polarity and intercellular interactions. Any disturbance may result in failure to join the developmental process leading to multiple types of facial clefts. 22 The importance of genomic research on the etiology of facial clefts is ever increasing. With advancements in the field of molecular biology, our envelope of research has grown. Identification of genetic polymorphisms in our population would be invaluable in understanding the developmental mechanisms involved in causing the disease. Data from animal models, in which clefts arise either spontaneously or as a result of mutagenesis experiment, combined with an analysis of how expression patterns correlate with gene function, and examining the effects of gene-environment interactions have proven themselves as powerful tools for identifying candidate genes for complex traits, like NSCLP. These animal model studies contribute to our knowledge of normal craniofacial development and the molecular pathogenesis of CLP, taking into account that facial development in mice mirrors human craniofacial development.

Research on various populations identified various candidate genes associated with CLP. Associations between SNPs in *RUNX2* , *BMP4* , *TGFB3* , *PAX7* , *NTN1* , *IRF6* , *PTHFR* , *GHR* , *CRISPLD2* , etc., and risk of clefts have been identified in different populations. Various genetic studies were conducted on diverse populations both on syndromic and nonsyndromic cases. Only IRF6 variants showed consistency in the etiology of CLP in different populations. Studies were conducted on case-parent trios, isolated clefts, and familial cases. In India, research on familial nonsyndromic cases is very less. Hence, the study was conducted on familial cases of NSCLP. Familial CLP corresponds to around 3.5% of the total cleft cases. 4 GSR Institute of Craniofacial surgery is a high-volume cleft center located in India. The sample population was taken from this center as patients from different parts of the country come for treatment. The nonsyndromic and familial cases were identified after a thorough medical history and examination of the patients.

*CRISPLD2* is an essential gene involved in protein-coding and reported to be associated with cleft lip palate in Chinese, 17 18 Irish population, 23 and studies on animal models. 19

Three polymorphisms, rs1546124, rs4783099, and rs16974880, were analyzed in the NSCLP as there is no reported literature on the role of these polymorphisms in the Indian population.

The results of the present study suggest that rs1546124, rs4783099, and rs16974880 are not significantly associated with NSCLP. In Northwestern Chinese population, SNP rs1546124 is significantly related to NSCLP whereas SNP rs4783099 was associated considerably to cleft palate only. 17 In Uyghur population, rs1546124 was associated with NSCLP. 18 In a meta-analysis, the authors concluded that rs4783099 induced a significantly increased risk for CLP. 24

The variation or inconsistent association of these polymorphisms for the different populations/ethnicities in the etiology of CLP could be due to multifactorial, the difference in the ethnicity, epigenetic causes, and gene-to-gene interactions. Future research should focus on the additional markers on *CRISPLD2* , incorporation of isolated cases of clefts, and also on the functional role of these SNPs.

## Conclusion

The results of this study indicate that among the multiplex families in India, the polymorphisms rs1546124, rs4783099, and rs16974880 of *CRISPLD2* not associated with increased risk of NSCLP. Further studies are required to study the role of other SNPs on this gene and other candidate genes with bigger sample size.
